# Comparison of the Adherence to the American Diabetes Association Guidelines of Diabetes Care in Primary Care and Subspecialty Clinics

**DOI:** 10.1186/s40200-015-0158-x

**Published:** 2015-04-24

**Authors:** Deepthi Takkalapelli Rao, Lily Kristine Sunio, Yun-Jia Lo, Ved Vyas Gossain

**Affiliations:** Division of Endocrinology and Metabolism, Michigan State University, 788 Service Road, Room B319, East Lansing, MI 48824-7016 USA; Division of Endocrinology, Michigan State University, 788 Service Road, Room B319, East Lansing, MI 48824-7016 USA; Present address: Division of Endocrinology, Michigan State University, 832 East Colfax Ave, Mishawaka, IN 46545 USA; College of Education, Michigan State University, Ann Arbor, MI USA; Present address: School of Natural Resources and Environment, University of Michigan, Ann Arbor, MI USA

**Keywords:** Diabetes mellitus, Diabetes care in primary care clinics, Diabetes care in subspecialized diabetes clinics

## Abstract

**Background:**

Diabetes mellitus is a major public health problem with significant morbidity and mortality. Evidence based guidelines have been proposed to reduce the micro and macrovascular complications, but studies have shown that these goals are not being met. We sought to compare the adherence to the American Diabetes Association guidelines for measurement and control of glycohemoglobin (A1c), blood pressure (BP), lipids (LDL) and microalbuminuria (MA) by subspecialty and primary care clinics in an academic medical center.

**Methods:**

390 random charts of patients with diabetes from Family Practice (FP), Internal Medicine (IM) and Diabetes (DM) clinics at Michigan State University were reviewed.

**Results:**

We reviewed 131, 134 and 125 charts from the FP, IM and DM clinics, respectively. DM clinic had a higher percentage of patients with type 1 diabetes 43/125 (34.4%) compared with 7/131 (5.3%) in FP and 7/134 (5.2%) in IM clinics. A1c was measured in 99%, 97.8% and 100% subjects in FP, IM and DM clinics respectively. B.P. was measured in all subjects in all three clinics. Lipids were checked in 97.7%, 95.5% and 92% patients in FP, IM and DM clinics respectively. MA was measured at least once during the year preceding the office visit in 85.5%, 82.8% and 76.8% patients in FP, IM and DM clinics respectively. A1C was controlled (<7%) in 38.9, 43.3, 28.8% of patients in the FP, IM and DM clinics, respectively (p = 0.034). LDL was controlled (<100 mg/dl or 2.586 mmol/l) in 71.8, 64.9, 64% of patients in the FP, IM and DM clinics, respectively. MA was controlled (<30 mg/gm creatinine) in 60.3%, 51.5% and 60% patients in FP, IM and DM clinics respectively (P = 0.032). BP was controlled (<130/80) in 59.5, 67.2 and 52.8% patients in the FP, IM and DM clinics, respectively.

**Conclusion:**

Testing rates for A1C, LDL, and MA were high, in both subspecialty and primary care clinics. However, the degree of control was not optimal. Significantly fewer patients in the DM clinic had A1c <7%, the cause of which may be multifactorial.

## Introduction

Diabetes mellitus is a major public health issue in the US. In 2010, 25.8 (8.3%) million persons had diabetes, out of which 7.0 million were undiagnosed [1]. This number has increased to 29 million in 2012, an increase of 13% [2]. Diabetes related annual healthcare expenses are estimated to be 245 billion dollars with the medical expenditure for a person with diabetes being 2.3 times higher than a non-diabetic [3]. Clinical trials have consistently demonstrated a decrease in micro vascular complications with optimal glycemic, blood pressure and lipid management [4-7] and possible reduction in macro vascular disease [8]. Evidence based guidelines have been proposed to decrease morbidity and mortality from diabetes [9,10]. The American Diabetes Association (ADA) publishes these guidelines annually, and these guidelines are widely followed in the primary care and in the specialized diabetes clinics. However, the adherence to these current practice guidelines has been less than optimal [11]. We, therefore, decided to investigate how well these ADA guidelines were being followed in our primary care clinics and also in the subspecialized diabetes clinic. Subspecialists in diabetes undergo extensive training in the management of diabetes and specialized diabetes centers often provide a multidisciplinary approach to diabetes care with provision of medical, dietary and other care under one roof. However, existing studies evaluating specialist vs. primary care in the treatment of diabetes have yielded inconsistent results [12-24]. We hypothesized that specialized diabetes clinics provide superior quality of care and better patient outcomes compared to primary care clinics. Our primary aim was to compare the adherence to American Diabetes Association guidelines for measurement and control of glycohemoglobin (A1c) blood pressure (BP), lipids (LDL) and microalbuminuria (MA) by Internal Medicine, Family Practice and subspecialty clinics in an academic medical center. The study began in November 2010 and was completed by August 2012.

## Methods

This study was approved by the Michigan State University and Sparrow Hospital Institutional Review Boards. ADA recommends that A1c should be determined at least two times a year in patients who are meeting treatment goals and who have stable glycemia control and quarterly in patients whose therapy has changed or who are not meeting glycemic goals. For blood pressure control, the ADA recommends that hypertension should be treated to a systolic blood pressure goal of <140 mmHg and a diastolic goal of <80 mmHg. Lower systolic blood pressure targets such as <130 mmHg may be appropriate for certain individuals, such as younger patients, if it can be achieved without undue treatment burden [10]. However, in the earlier versions of the ADA guidelines, the recommended blood pressure target was <130/80 [9], and we have used that level to determine if blood pressure was controlled or not. For lipid levels, the ADA recommends that, individuals without overt cardiovascular disease, the goals should be: LDL cholesterol <100 mg/dL, HDL cholesterol >50 mg/dL for women and >40 mg/dL for men and triglycerides <150 mg/dL [10].

### Study population

390 randomly selected charts of adult patients with diabetes from diabetes (DM), Internal Medicine (IM) and Family practice (FP) clinics at Michigan State University (MSU) were retrospectively reviewed. The DM clinic is located in one of our affiliated hospitals and provides subspecialty care to a referral population of approximately 1 million around Lansing, the capital city of Michigan. Diabetes care at this center is delivered by 2 board certified endocrinologists (who are also full time faculty members of the MSU Department of Medicine), 3 endocrinology fellows, 2 nurse-practitioners, and 2 dietitians. All non-physician health care providers at the center are certified diabetes educators. Patients at the DM clinic are typically seen only by referral from another physician and are transferred back to their primary care physician when their diabetes care is optimized. The IM and FP clinics are located in the outpatient facilities of MSU and cater to patients with a variety of illnesses including diabetes. Providers at the Internal Medicine clinic and Family Practice clinics are board certified internists, family practitioners as well as resident physicians who work under the supervision of an attending physician.

### Data collection

Retrospective review was conducted on 390 randomly selected patient charts at the 3 clinics from November 2010 to August 2012. We used a standardized chart abstraction form to collect and record data. Two of the authors (DR and LS) abstracted the charts. Data collected included age, gender, race, BMI, smoking status, type of diabetes, self-glucose monitoring, Metformin, other antidiabetic medications, insulin, aspirin, ACEi (Angiotensin Converting Enzyme Inhibitors), angiotensin receptor blockers (ARBs), and statin use. We also assessed the measurement and control of A1C, LDL, BP and microalbuminuria during the one year period preceding their most recent visit. The laboratory data (A1C, lipid levels, and microalbuminuria) collected were done as part of routine clinical care of the study subject and were done at local clinical laboratories. Data was collected retrospectively for up to 12 months prior to the most recent visit for each patient.

### Statistical analysis

Data was analyzed to determine the average age, BMI of the patient population, gender distribution, percentage of smokers, type 1 vs type 2 diabetics and also if self-glucose monitoring was being performed. We also analyzed the proportion of patients taking Metformin, insulin, aspirin, ARBs, ACEi, statins at the time of the most recent visit. We analyzed the data to determine the measurement and degree of control of A1c, BP and lipids and microalbuminuria. Results are reported in percentages. Descriptive statistics were computed as means and standard deviations for continuous variables and as frequencies for categorical variables. We used ANOVA for continuous variables and chi square test for dichotomous variables. Logistic regression models were used to assess the factors affecting binary dependent variables. *P* values less than .05 were considered significant. The data analysis was performed by an independent biostatistician at the Michigan State University Center for statistical training and computing, using SPSS, version 17.

## Results

Results were obtained from 390 patient charts of which 131 were from the family Practice clinic (FP), 134 from the Internal Medicine clinic (IM) and 125 from the specialist diabetes center clinic (DM).

### Demographics

Baseline characteristics are shown in Table 1. Patients in the DM clinic with a mean age of 52.82 ± 16.02 were significantly younger compared to the patients in other two groups. There was no statistically significant difference between clinics as it relates to the gender distribution, smokers or those with a BMI > 30 kg/m^2^.

**Table 1:** **Baseline characteristics**

***Variable***	***FP*** ***(n = 131)***	***IM*** ***(n = 134)***	***DM*** ***(n = 125)***	***P value***
*Mean age*	61.00 ± 11.00	62.11 ± 12.88	52.82 ± 16.02	<0.001*
*Male*	74 (56.5%)	61 (45.5%)	72 (57.6%)	0.095
*Female*	57 (43.5%)	73 (54.5%)	53 (42.4%)	
*BMI >30*	71 (54.1%)	66 (49.2%)	58 (46.4%)	0.092
*Smoker*	14 (10.7%)	18 (13.4%)	21 (16.8%)	0.306
*Type 1*	7 (5.3%)	7 (5.2%)	43 (34.4%)	<0.001*
*Type 2*	124 (94.7%)	127 (94.8%)	82 (65.6%)	
*Self glucose monitoring*	58 (45.0%)	67 (50.0%)	86 (68.8%)	<0.001*
*Metformin*	107 (81.6%)	85 (63.5%)	43 (34.4%)	<0.001*
*Insulin*	34 (26.1%)	39 (29.1%)	99 (79.2%)	<0.001*
*Aspirin*	104 (79.4%)	84 (62.7%)	67 (53.6%)	<0.001*
*ACEI/ARB*	105 (80.2%)	105 (78.4%)	97 (77.6%)	0.876
*Statin*	92 (70.2%)	87 (64.9%)	81 (64.8%)	0.569

Age is given as mean ± SD.

Other values are absolute numbers with percentage in ( ).

*Statistically significant.

There were significantly greater number of type 1 diabetes patients in the DM clinic (34.4%) compared to the FP (5.3% and IM (5.2%) clinics (p < 0.01). Similarly insulin use (79.2%) and self glucose monitoring (68.8%) were higher in the DM clinic (p < 0.01). Use of aspirin (53.6%) and Metformin (34.4%) was significantly less among the DM clinic patients (p < 0.01). There was no significant difference in the use of Angiotensin converting Enzyme Inhibitors (ACEi)/Angiotensin Receptor Blockers (ARBs) or statins between the three clinics.

### Control of diabetes parameters

#### Hemoglobin A1c

Hemoglobin A1c was measured at least once in the preceding 6 months prior to the last visit in 99.2% of the patients in FP clinic, 97.8% of patients in IM clinic and 100% of the DM clinic patients. Significantly fewer patients (28.8%) in the DM clinic had their A1c controlled (less than 7%) compared to 38.9% in the FP clinic and 43.3% in the IM clinic (p = 0.034) (see Table 2).

**Table 2:** **Control of diabetes parameters**

***Variable***	***FP*** ***(n = 131)***	***IM*** ***(n = 134)***	***DM*** ***(n = 125)***	***P value***
*A1c Measured*	130 (99.2%)	131 (97.8%)	125 (100)	
*Controlled <7%*	51 (38.9%)	58 (43.3%)	36 (28.8%)	0.034
*BP Measured*	131 (100%)	134 (100%)	125 (100%)	
*Controlled <130/80)*	78 (59.5%)	90 (67.2%)	66 (52.8%)	0.062
*LDL Measured*	123 (93.9%)	123 (91.8%)	112 (89.6%)	
*Controlled <100*	94 (71.8%)	87 (64.9%)	80 (64%)	0.552
*Microalbumin Measured*	112 (85.5%)	111 (82.8%)	96 (76.8%)	
Controlled**	79 (60.3%)	69 (51.5%)	66 (60%)	0.032*

Values are absolute numbers with % in ( ).

*P < .05 was considered significant.

**Defined as <30 mg/gm creatinine.

#### Blood pressure

Blood pressure was recorded in all patients in the three clinics. There was no significant difference in blood pressure control (less than 130/80) between the three clinics (p = 0.062) with 59.5% of FP clinic patients, 67.2% of IM clinic patients and 52.8% of DM clinic patients being under control (see Table 2).

#### Lipids

Lipids were checked at least once in 97.7%, 95.5% and 92% of the FP, IM and DM clinic patients respectively. LDL level was at goal (less than 100 mg/dl or 2.586 mmol/l) in 71.8% of FP; 64.9% of IM and 64% of DM clinic patients (p = 0.552). (Mean ± SD), LDL level was 83.97 (29.91); 88.51 (34.17) and 87.21 (26.86) mg/dL in FP, IM and DM clinics respectively. Fifty seven percent, 59.1% and 68.7% of the FP, IM and DM clinic patients respectively had their triglycerides at target (less than 150 mg/dL). HDL was controlled (more than 50 mg/dL for women and more than 40 mg/dL for men) in 38.9%, 45.5% and 48.8% of the FP, IM and DM clinics respectively (see Table 2).

#### Microalbumin

Microalbumin was measured at least once during the year preceding the office visit in 85.5% of FP clinic patients, 82.8% of IM clinic patients and 76.8% of DM clinic patients (see Table 2).

### Factors affecting the control of diabetes care

A multivariate statistical analysis controlling for age, gender, race, BMI, smoking status, and type of diabetes to predict effects on A1c, lipids, B.P and microalbuminuria was performed (see Table 3).

**Table 3:** **Results of logistic regression models showing odds ratios**

	**A1c**	**BP**	**Lipid**	**Micro-albuminuria**
	**Controlled**	**Uncontrolled**	**Controlled**	**Controlled**
	**OR**	**OR**	**OR**	**OR**
Gender (ref. Female)				
Male	1.38	1.15	1.56*	1.12
AGE	0.99	0.99	1.03**	0.99
BMI	1.01	0.97**	1.01	1.02
Smoker (ref. Ex-smoker)				
Smoker	1.17	1.10	1.35	0.874
Non-smoker	0.97	1.39	0.88	0.094
Not Documented	1.13	2.32	0.65	1.646
Diabetes (ref. Type 1)				
Type 2	1.37	1.46	0.93	0.46
Self-Glucose Monitor (ref. No) Yes	1.00	NA	NA	2.59***
Not Documented	0.80			1.62
Insulin (ref. No)		NA	NA	NA
Yes	4.37***			
LDL Cholesterol (ref. = < 100)	NA		NA	
>100		1.89**		0.95
ACE inhibitor (ref. Not taking )	NA		NA	
Took		0.70		0.57
ARB (ref. Not taking )	NA		NA	
Took		0.59		0.74
Beta blocker (ref. Not taking )	NA		NA	NA
Took		0.86		
Diuretic (ref. Not taking )	NA		NA	NA
Took		0.54*		
CCB (ref. Not taking)	NA		NA	NA
Took		0.73		
Number of Medicine Taken	NA	1.10	NA	0.77**
BP (ref. control = <130/80)	NA	NA		
Uncontrolled > 130/80			0.56**	0.94
A1c (ref. Uncontrolled)	NA	NA	NA	
control				0.94

Note: Level of significance: ***p-value < .001; **p-value < .05; *p-value < .1.

CCB = Calcium channel blockers.

For A1c, additionally insulin use and self glucose monitoring was also included in the model and the analysis revealed that insulin use was the only significant factor affecting A1C control. Insulin use was associated with poor glycemic control. Patients on insulin were more likely to have A1C ≥7% (OR = 4.37, p = 0.01).

With regard to BP, additionally, LDL control, use of antihypertensive drugs and the total number of drugs used was included in the model. The analyses revealed that patients on diuretics, were more likely to have controlled B.P (<130/80) (OR = 1.7, p = 0.05) and those with LDL > 100 were more likely to have uncontrolled BP (>130/80) (OR = 1.89, p = 0.02).

There was also an association of age with controlled LDL levels in that older subjects were more likely to have LDL >100 mg/dl (OR = 1.03, p = 0.01).

For microalbuminuria, the additional controlled factors were use of ACEi/ARBs, total number of antihypertensive drugs A1c, lipids, self glucose monitoring and control of B.P. Self glucose monitoring was associated with controlled microalbuminuria (<30 mg/gm creatinine; OR =2.59, P < 0.01). Subjects taking increased number of antihypertensive drugs were less likely to have controlled microalbuminuria (OR = 0.77, p < 0.01).

## Discussion

The main findings of our study are that the ADA guidelines are being adhered to in our primary care (FP, IM) and diabetes specialty clinic. We have also evaluated the degree of control of some of the diabetes care parameters and compared our results to the data presented by Ali et al. [11] where they analyzed the quality of diabetes care in the US from 1999 to 2010.

In our study, A1c was measured in 97.8 to 100% of the patients depending on the clinic; however, only 28.8 to 43.3% of the patients achieved a target A1c below 7%. This is lower than that reported by Ali et al. [11] during the time period of 2007 to 2010, where 52.2% subjects were reported to have an A1c <7%. They also reported that the glycemic control defined as A1c <7% improved by 9.4 percentage points (95% CI 3.0-15.8 from the years 1999–2010.

The ADA guidelines from 2010 [9] which were the standards of care during the duration of this study recommend a blood pressure target less than 130/80 mm of Hg. In our study, BP was measured 100% of the time in all the patients, but controlled in 52.8% to 67.2% of patients among the three clinics. This is higher than the reported rate of BP control in diabetics in the US (51.3%) during 2007 to 2010 [11]. Nationally the blood pressure control improved by 11.7 percentage points (95% CI 5.7-17.7) from 1999–2010.

The ADA recommends checking a fasting lipid profile at least annually and every 2 yearly in those with controlled values. In our study, lipid profile was checked at least once annually in 89.6 to 93.9% of the patients based on the clinic. LDL was controlled in 64 to 71.8%, triglycerides were controlled in 57 to 68.7% of the patients and HDL was controlled in 39.8 to 53% of the patients. Our LDL control rates appear to be better than the 56.8% control rate of the national diabetes population in the US, for years 2007–2010 [11]. Again, nationally the control of LDL, defined as <100 mg/dL improved by 20.8 percentage points (95% CI 11.6-30.0) between 1999 and 2010. In our study, 76.8 to 85.5% patients had their micro albumin measured but microalbuminuria was controlled in 51.5% patients in the IM clinic. This difference was statistically significant (p = 0.032) compared to the other two groups where the microalbuminuria was controlled in 60% patients. The rate of controlled microalbuminuria is lower than that reported rate in the US diabetic population during 2007 to 2010, where 69.8% of survey participants were free of microalbuminuria (<30 mg/gm creatinine).

There were no significant differences in the gender distribution, number of smokers and obese between the three clinics in our study. With regards to age, the DM clinic population in our study was significantly younger compared to the FP and IM clinics. This is consistent with the findings of several other studies [13-18]. This could be explained by the constitution of higher number of type 1 diabetics in the subspecialty clinics. Our study results show significantly higher number of type 1 diabetics in the DM clinic similar to the study by Grant et al. [18] Insulin use was significantly higher in the DM clinic in keeping with the findings of several other studies [14,16,18]. We also found significantly increased rate of self-glucose monitoring by patients of the DM clinic similar to the findings of Zgibor et al. [19].

We found that ADA guidelines are being followed in the majority of our study population in the monitoring of A1c, blood pressure, lipids and urine albumin excretion. However, the degree of control of these parameters is not optimal. The number of patients with an A1c lower than 7 was significantly lower in our DM clinic compared to IM and FP clinics. There was no significant difference in the control of BP and lipids, but the control of MA was significantly lower in the IM clinic.

Several studies comparing specialist and general practitioner care in diabetes failed to show a consistent superiority of specialist care in diabetes outcomes. In results from the Medical Outcomes Study, Greenfield et al. [20] compared glycemic and BP control, visual function, foot ulcers and albumin excretion rate among endocrinology, general internist and family practice clinics, there was no significant difference in quality measures based on physician specialty except improved foot ulcer prevalence in endocrinologist care. No difference in adjusted outcome and process measures was found between specialist and primary care in another study by Greenfield et al. [14]. Similarly, Ismail et al. [21] conducted an observational study which showed that patients seen in specialist clinics had a higher A1c at baseline than those receiving routine primary care, but there was no difference in the rate of improvement with each visit.

There are also other studies demonstrating that specialty care leads to better performance in diabetes management. A retrospective study by Ho et al. [12] comparing specialist and generalist care in the VA setting found that the patients under specialist care had better processes of care including recording of A1c, blood pressure, foot and eye exams, but there was no measurement of outcomes in this study. Zgibor et al. [19] studied type 1diabetic participants in the Pittsburgh Epidemiology of Diabetes Complications Study and found significantly lower mean A1c levels in those receiving specialist care. In a long term follow up study from the same population they also found that prior specialist use was significantly protective against the development of overt nephropathy, neuropathy and weakly protective against coronary artery disease [13]. Suwattee et al. [15] found that diabetes clinic performed better in A1c, BP and LDL outcomes compared to resident and faculty care in general medicine clinics. Although process measures were significantly more in favor of specialist care in a study by De Berardis et al. [16] there was no difference in diabetes outcomes except total cholesterol. Uchigata et al. [22] studied type 1 diabetics in Japan and found that those receiving specialist care were significantly less likely to develop end stage renal disease and less likely to die. In a study by Grant et al. [18]. Endocrinology clinic had better A1c, BP and LDL values and urine albumin screening. Shah et al. [17] showed better A1c values in specialist care while Sone et al. [23] found that baseline A1c values significantly improved under specialist management. In terms of health care expenditure, Levetan et al. [24] showed that for patients admitted with diabetic ketoacidosis, Endocrinologist care reduced the length of stay, hospital expenditure and readmission rate.

Although, from our study, it appears that the glycemic control is worse in a specialist clinic, there are several factors which could explain this. It could be due to referral bias, wherein, patients who are referred to specialist care are poorly controlled when they are referred. Patients seen by specialists are usually under the care of multiple physicians leading to fragmentation of care and deferment of some services to other providers. Specialists are also more likely to individualize glycemic targets depending on co morbid conditions and therefore assessing control based on A1c less than 7% may not be valid for the complexity of patients seen at the DM clinic. Our data also does not reflect the duration of specialist care for the patients. In the management of a chronic condition likely diabetes, long term follow up is needed to assess improvement in outcomes. This is especially relevant since longitudinal follow up studies [13,16,21,22] of patients under specialist care have shown improvement in diabetes parameters and outcomes over time. Also, it has been the practice of our DM clinic to return the patients who have achieved stable and improved glycemic control back to their primary care providers. Therefore, patients who are not well controlled remain in the DM clinic and this may partly explain the presence of fewer patients with A1c <7%.

Our study does have limitations. While a randomized controlled trial is the ideal type of study to measure the impact of an intervention, ours is a retrospective observational study. Our data only reflect the measures at a single point. We do not have follow up data and therefore it is not possible to gauge change over time. We do not have information on all the comorbidities of the patients which can affect their metabolic control and glycemic targets. Our study also does not include data about patients receiving care from other specialists, which may lead to physicians’ inaction with regards to medication adjustment for uncontrolled blood pressure and lipids.

## Conclusion

In our study the specialist clinic patients were younger with a higher proportion of type 1 diabetics and insulin users. There were no significant differences in the gender and weight distribution, smoking status, BP and LDL control. Although A1c appeared better in FP and IM clinics compared to DM clinic, the nature of the study precludes the inference that there is no difference between diabetes care provided in a specialty center vs. a general clinic, although other studies have shown improvement in diabetes parameters with specialist care, but unequivocal evidence of improved mortality is lacking. Further long term studies to assess the impact of subspecialty care on diabetes are warranted.
